# Aberrant Notch signaling in gliomas: a potential landscape of actionable converging targets for combination approach in therapies resistance

**DOI:** 10.20517/cdr.2022.46

**Published:** 2022-10-18

**Authors:** Maria D’Amico, Francesca De Amicis

**Affiliations:** ^1^Department of Pharmacy, Health and Nutritional Sciences, University of Calabria, Via P. Bucci, Rende 87036, Italy.; ^2^Health Center, University of Calabria, Via P. Bucci, Rende 87036, Italy.

**Keywords:** Tumor microenvironment, notch inhibitors, stem cells, brain tumors, drug resistance

## Abstract

The current therapeutic protocols and prognosis of gliomas still depend on clinicopathologic and radiographic characteristics. For high-grade gliomas, the standard of care is resection followed by radiotherapy plus temozolomide chemotherapy. However, treatment resistance develops due to different mechanisms, among which is the dynamic interplay between the tumor and its microenvironment. Different signaling pathways cause the proliferation of so-called glioma stem cells, a minor cancer cell population with stem cell-like characteristics and aggressive phenotype. In the last decades, numerous studies have indicated that Notch is a crucial pathway that maintains the characteristics of resistant glioma stem cells. Data obtained from preclinical models indicate that downregulation of the Notch pathway could induce multifaceted drug sensitivity, acting on the expression of drug-transporter proteins, inducing epithelial–mesenchymal transition, and shaping the tumor microenvironment. This review provides a brief overview of the published data supporting the roles of Notch in drug resistance and demonstrates how potential novel strategies targeting Notch could become an efficacious action to improve the therapy of high-grade glioma to overcome drug resistance.

## INTRODUCTION

The most frequent malignant primary brain tumor in adults is glioma. Low-grade glioma (LGG) includes WHO grades 1-2, while high-grade glioma (HGG) includes WHO grades 3-4. Glioblastoma (GBM), one of the most lethal and most prone to recurrence of malignant solid tumors, represents the majority of WHO grade 4. GBM accounts for more than 50% of all gliomas and primary malignant brain tumors^[1]^. Current molecular diagnostic techniques allow alternative classification based on molecular abnormalities and signaling pathways involved in glioma genesis. Isocitrate dehydrogenase (IDH) is a basic biomarker for subtyping and prognosis of gliomas. Tumors that are IDH-mutant have a better prognosis than their counterparts with similar histologic grade and IDH-wildtype phenotype^[2]^. Furthermore, methylation of the O6-methylguanine DNA methyltransferase (MGMT) promoter is related to a better response to chemotherapy with temozolomide (TMZ) and longer overall survival^[3]^. However, the combination of multiple tumorigenic events, such as highly deregulated tumor genome with hyper-activation of oncogenes, causes and sustains the pathogenesis of glioma and resistance to various therapies^[4]^. For instance, oncogenic activation of receptor tyrosine kinases (RTKs) is a crucial signaling mechanism for maintaining glioma oncogenicity. Among all the RTK alterations, epidermal growth factor receptor (EGFR) gene amplification is the most common variation (approximately 40%) in GBM, although EGFR inhibitors (e.g., gefinitib and erlotinib) have not caused clinical effects in patients with GBMs in clinical trials^[5]^. Increased expression of platelet-derived growth factor receptors (PDGFR) and PDGF has been evidenced in astrocytic cancers of different grades and is associated with poor prognosis^[6]^. Although GBM patients present sporadic RAS alterations, RAS and AKT mediate fundamental pathways in different models of GBM^[7]^. In addition, Sonic Hedgehog homolog (SHH), which mediates molecular signals to the embryonic cells required for normal development and Notch, plays a crucial role as an important factor influencing neural progenitor behavior and is dysregulated in glioblastoma stem cells (GSCs)^[8]^. In the next sections, we recapitulate the state of our knowledge regarding the functional role of the Notch signaling pathway, focusing on GBM disease progression and therapy resistance and suggesting novel treatments to overcome drug resistance for effective therapeutic protocols of brain malignancies.

## NOTCH SIGNALING

The Notch receptor mediates a highly conserved signaling pathway that is crucial in central nervous system development and malignant transformation^[9,10]^. Four Notch receptors (Notch1 to Notch4) interact with different canonical activators such as delta/serrate/lag-2 (DSL) ligands Jagged1 and Jagged2 and delta-like (Dll) 1, Dll3, and Dll4 of the Notch pathway^[11]^. Notch1 and Notch2 are extensively expressed in numerous tissues during embryonic development and in adult mammals^[12]^. Vascular smooth muscle and pericytes express elevated Notch3, while Notch4 is much more abundant in the endothelium^[13,14]^. Notch receptors, located on the cell membrane^[15]^, are characterized by N-terminal EGF repeats that compose the extracellular portion and by a juxtamembrane negative regulatory region (NRR). The intracellular region is formed by a transcriptional activation domain and a C-terminal degron domain rich in the amino acids proline, glutamate, serine, and threonine (PEST)^[13,16]^. Notch signaling is initiated by the contact of DSL ligands from sending cells and Notch receptors existing on proximal cells. After this occurrence, the Notch protein undergoes additional proteolytic cleavage determined by the γ-secretase enzyme complex, originating in the Notch intracellular domain (NICD) which translocates into the nucleus. NICD binds to DNA/chromatin-specific sequences, displaces a repression complex, interacts with the DNA binding protein recombination signal binding protein for immunoglobulin kappa j region (RBPJ), and recruits the co-activator mastermind-like1 (MAML1)^[17]^. Next, the ternary complex recruits the histone acetyltransferases P300/CBP-associated factor (PCAF), a key regulator of transcription which is expressed in GBM cells^[18]^. Lastly, these protein complexes induce the expression of Notch-regulated genes, for example, hairy enhancer of split (Hes) and HES-related proteins (Hey), both important in lineage-commitment choices. In addition, cell cycle regulators such as p21/Waf1, CYCLIN D1 (CD1) and CD3, and an important regulator of stem cell biology (c-Myc), an EGFR-related gene found to be amplified in human breast cancer cell lines such as the human epidermal growth factor receptor 2 (HER2), as well as nuclear factor kappa B (NF-κB), insulin-like growth factor 1-receptor (IGF1-R), survivin, snail homolog 2 (SLUG), SOX2, and paired box (PAX) 5^[19,20]^, all directly associated with tumorigenesis, are described as Notch target genes^[20,21]^. The Notch-dependent transcriptional activity concludes with NICD degradation. Cyclin-dependent kinase 8 (CDK8), which binds to and/or phosphorylates several transcription factors, causes the phosphorylation of a degron within the PEST domain of NICD and is targeted for proteasome-mediated degradation by E3 ubiquitin ligases SEL10 (FBW7)^[15]^. The Notch pathway may be regulated through post-translational modification^[22]^, which changes the affinity of the Notch receptor for DSL. The stability of NICD and the extent of signaling are also influenced by phosphorylation of Notch proteins induced by glycogen synthase kinase 3β (GSK3β), largely expressed throughout the brain with an additional expression on endothelial cells in mice^[23]^. Beyond the established signaling stimulation, distinct proteins missing the DSL domain have been described^[24]^, such as membrane-integral proteins delta/Notch-like epidermal growth factor-related receptor (DNER), a glycosylphosphatidylinositol (GPI)-linked membrane (e.g., NB3/Contactin6), or secreted proteins^[25]^. For instance, NICD physically cooperates with a major component of canonical WNT signaling, β-catenin^[26]^, Smad tumor suppressor proteins^[27]^, and the key regulators of cellular response to changes in oxygen concentration, such as hypoxia-inducible factor 1 alpha (HIF-1α)^[28]^, thus obtaining direct crosstalk between Notch and the wingless/integrated (WNT), transforming growth factor-beta (TGF-β), and hypoxia-dependent signaling pathways.

## NOTCH SIGNALING IN CANCER

Given the outstanding position of Notch in regulating cellular behavior, it is well recognized that Notch signaling is altered in a wide range of diseases, including human malignancies. Unbiased genome-scale sequencing data indicate mutations in Notch genes in various types of cancers. Interestingly, the positions, identities, and effects of these mutations represent varied roles for Notch in different types of cancers^[29]^, such as breast cancer^[30]^, prostate cancer^[31]^, lung cancer^[32]^, glioblastoma^[33]^, and other malignancies^[34]^. Definitely, functional studies consider Notch signaling one of the hallmarks of cancer, although several data suggest both oncogenic and tumor suppressive functions, depending on the cancer type.

Three separate patterns of Notch gene mutations have been highlighted in several malignant tumors: (1) strong gain-of-function mutations that disrupt the NRR, with or without PEST degron domain deletions, as evidenced in T cell acute lymphoblastic leukaemia (T-ALL) and triple-negative breast cancer (TNBC); (2) PEST degron domain deletions only, as shown in B cell tumors; and (3) disruptive nonsense, frameshift, or point substitutions in the N-terminal portions of Notch receptors, which probably lead to loss of Notch function, as seen in squamous cell carcinoma, small cell lung carcinoma, bladder carcinoma, and certain low-grade gliomas^[16]^. A recent study focused on the frequent RBPJ copy number loss and diminished RBPJ protein expression in a significant minority of several types of carcinoma, mainly breast carcinomas^[35]^. Nevertheless, the connection between changed RBPJ gene dosage and tumorigenesis is multifaceted and may diverge depending on the tumor type.

The diverse mutational patterns of Notch receptor genes in specific cancers suggest that Notch can function as oncogenic signaling as well as a tumor suppressor in other situations^[13]^. The initial demonstration of an onco-suppressor action of Notch comes from results obtained in keratinocytes from mice and humans^[36]^. More recent data from whole-genome sequencing analysis describe Notch-inactivating mutations in patients with head and neck squamous cell carcinoma^[37]^. Thus, Notch signaling mediates both oncogenic and onco-suppressive action in similar tissue, as demonstrated in the hematopoietic system. Such a dual role is related to the action exerted by Notch in the modulation of cell fate decisions in immune cell expansion.

In addition, experimental data establish that the Notch signal has a fundamental part in cancer patient survival. For example, high expression of Jagged1 or Notch1 correlates with poorer overall survival when compared with low levels of both. Jagged1 was also found to be highly expressed in metastatic prostate cancer compared to localized prostate cancer or benign prostatic tissues^[38,39]^. High levels of Jagged1 and Notch1 have been detected in breast cancers and were related to poor prognosis^[38]^. Shi *et al.* similarly reported that the Notch expression profile in papillary bladder transitional cell carcinoma appears dissimilar to the matched invasive phenotype. Consequently, Notch1 and Jagged1 may be used as additional markers for the prognosis of papillary bladder transitional cell carcinoma patients^[40]^. Cervical carcinomas patients positive for nuclear Notch3 expression showed shorter overall survival when compared with Notch3-negative patients; thus, Notch3 is a possible prognostic marker in cervical carcinomas^[41,42]^. Taken together, these data fuel additional scientific interest regarding the investigation of the Notch family as diagnostic and prognostic markers in human cancer.

## NOTCH SIGNALING IS ABERRANTLY ACTIVATED IN BRAIN TUMORS

In recent years, large-scale genomic sequencing on brain tumors provided a unique understanding of the genomic aberrations and cellular signaling mechanisms that control brain tumor initiation and progression^[43]^. Among the different pathways, aberrant Notch signaling was found in brain tumors, even though its members are infrequently altered^[43]^. Elevated Dll1, Notch1, Notch3, Notch4, and HEY1 levels were associated with advanced glioma grade and poor prognosis^[44,45]^, strongly suggesting that Notch signaling influences an undifferentiated and aggressive brain cancer phenotype. mRNA and protein levels of Notch1, Notch4, Dll1, Dll4, Jagged1, HEY1, HEY2, and HES1 are increased in brain tumors and associated with upregulation of vascular endothelial growth factor (VEGF) and phospho-AKT, together with a reduction of the tumor suppressor phosphatase and tensin homolog (PTEN)^[33,46]^. However, data suggest a controversial role of Notch1 in glioma genesis^[47]^. Specifically, Notch1 levels are increased in patients with a survival of > 1 year compared to < 1 year^[48]^. Thus, outstanding questions remain to be addressed. A very recent study suggested the Notch canonical ligand, Dll3, as a key therapeutic target and/or prognostic marker in LGG. The authors demonstrated that Dll3 promoter methylation and decreased mRNA expression influenced the patient’s prognosis in LGG but not in GBM. This is a crucial discovery that identified subsets of LGG with different immune microenvironments which depend on Dll3 mRNA expression^[49]^.

### Notch signaling in glioblastoma drives GSCs phenotype

GBM harbors various cellular categories, several with amplified aggressiveness and stemness of GSCs, which are responsible for tumor relapse and therapy resistance^[50,51]^. GSCs are regulated by genetic, epigenetic, and metabolic factors, in addition to extrinsic factors, the immune system, and the tumor microenvironment (TME)^[52]^. Indeed, vascular cells, microglia, peripheral immune cells, and neural precursor cells that populate the TME control the course of the disease^[53]^. Specifically, the multiple molecular interactions of bulk glioma cells and GSCs with the TME exert a pathological impact influencing tumor progression and response to different treatments^[53]^. These observations suggest targeted therapeutic approaches that are effective against heterogeneous GBM cell populations^[54]^.

**Figure 1:**
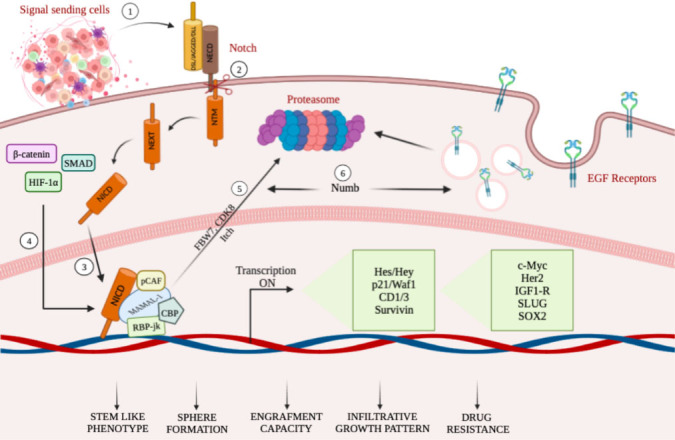
A schematic summary illustrating mechanisms targeting Notch in GCSs (see the text for details). (1–3) TME cell populations, secreting a number of factors including Notch ligands, stimulate the transcriptional activity of Notch receptors, thus influencing tumor biology. (4) NICD physically cooperates with β-catenin, Smad proteins, and HIF-1α, thus obtaining the crosstalk among Notch, Wnt, TGF-β, and hypoxia-dependent signaling pathways. (5) Notch-mediated transcriptional activation achieves the NICD degradation through phosphorylation of NICD, mediated by CDK8 and targeted for proteasome-mediated degradation. (6) Decrease of EGFR expression due to Numb-mediated endocytosis.

The potent tumorigenic activity of GSCs has been associated with the expression of Nestin, glial fibrillary acidic protein (GFAP), β-III tubulin, and CD133 stem cell markers^[55]^. Among the different aberrant signaling pathways, the Notch pathway, co-opted in GBM, stimulates astrocytes to assume a stem-like state through different mechanisms (as summarized in Figure 1), which can be accompanied by increased proliferation^[56]^. The dependence of GSCs on Notch signaling is further supported by experiments demonstrating depletion of GSCs by treatment with γ-secretase inhibitors^[57]^. Interestingly, cell fate determinant Numb4 controls the expression of stem cell markers in GSCs, which function as regulators of Notch signaling. Overexpression of Numb4 decreased EGFR expression due to Numb-mediated endocytosis^[58]^.

Active Notch signaling is essential for maintaining the tumorigenic potential of GSCs and drug resistance under hypoxic conditions. Accordingly, stimulation of the Notch pathway by HIF-1α in GSCs is crucial for hypoxia-mediated effects. Indeed, reduction of HIF-1α or inhibition of Notch signaling partially prevents the hypoxia-mediated maintenance of GSC^[59]^.

Notch effects are also evident in terms of metabolic reprogramming, which is mainly influenced by mitochondrial respiration. Reverse electron transfer (RET), which influences ROS levels under a variety of metabolic conditions, many of which are associated with pathology, is particularly active in GCSs. Notch modulates RET through the interaction with specific respiratory chain complex I (RC-I) proteins. The authors demonstrated that genetic and pharmacological interference of Notch and RET inhibited GSC proliferation in mouse models. These results demonstrate the crucial role of Notch as a regulator of metabolic reprogramming of brain tumors, suggesting novel therapeutic purposes^[60]^ targeting the metabolic regulation of GBM cells. Besides, further data support the multifaceted role of Notch. On the one hand, Notch2 expression levels are associated with Nestin and SOX2 stemness markers, together with vimentin and GFAP, as well as anti-apoptotic proteins, but they are also inversely associated with pro-apoptotic proteins in GBM tissue^[33]^. On the other hand, several groups have reported dissimilar expressions of Notch1, Notch2, MAML1, and pPCAF300 in human glioma, with controversial results regarding tumor progression and prognosis^[47]^.

### Notch Signaling drives drug resistance and disease progression dependent on GSCs

**Figure 2:**
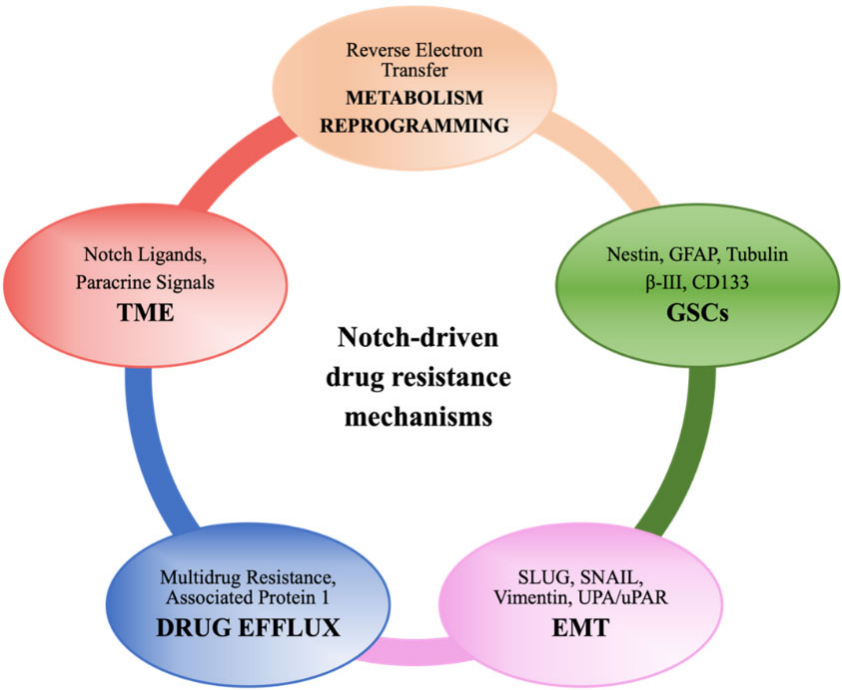
A schematic illustration showing Notch-driven drug resistance mechanisms (see the text for details).

In GBM, innate and acquired mechanisms of treatment resistance could depend on the blood - brain barrier, tumoral heterogeneity, and TME. In addition, GSCs are actually retained to support GBM progression and resistance to different drugs^[61]^. Different mechanisms have been described, and, more recently, a live-imaging study of GSCs acquired from the infiltrative area and putative relapse-driving area showed that GSCs are interconnected and able to transfer mitochondria via thin membranous open-ended channels, connecting distant cells. The authors concluded that this novel mechanism might have a potentially relevant role in therapy resistance; however, the increased stem cell population, DNA repair activity, expression of multidrug transporters, and redundant signaling pathways such as Notch^[62]^ are better established and investigated mechanisms of resistance (as summarized in Figure 2). Data from different experimental models suggest that anticancer drugs such as TMZ^[63]^, bevacizumab^[64]^, and oxaliplatin^[65,66]^ frequently induce Notch, which in turn controls the factors implicated in drug efflux, metabolic reprogramming, regulation of GSCs and TME, etc. that lead to acquired resistance. Loss of serine/threonine-protein kinase polo-like kinase 2 (PLK2), a key regulator of centriole duplication, was found to be strongly related to acquired resistance to TMZ via stimulation of the Notch pathway in GBM. Particularly, the authors suggested that loss of PLK2 induced the Notch pathway, mediated by the transcriptional inhibition of HES1^[63]^.

Several fundamental mechanisms have been described; for example, Notch1 regulates the expression of multidrug resistance-assosicated protein 1 (MRP1), which plays an intrinsic role in the chemoresistance of GBM tumor cells^[67]^; thus, Notch is an encouraging target for the improvement of the current glioma therapeutic portfolio. Blocking Notch signaling or RBPJ reduced clonogenic potential in tumor-sphere assays and engraftment capacity in GBM xenograft models^[68]^. Accordingly, NICD overexpression could induce cell survival due to radioresistance and side population phenotype in glioma cells^[69]^. Moreover, the extracellular matrix glycoprotein Tenascin-C and Jagged1 strengthen the expression of each other, promoting brain tumor-initiating cell (BTIC) growth. These cells from human GBM patients exhibit increased resistance to radiation and chemotherapy^[70]^.

GSC population enrichment and its increasing resistance to treatment are favored by the brain TME, secreting a number of factors including Notch ligands which influence tumor biology. For instance, endothelial cells provide ligands, bind the Notch receptors, and are expressed by GSCs. Paracrine signals from mesenchymal stem cells (MSCs) induce chemosensitization to TMZ in heterogeneous GSCs that lack EGFR amplification through the downregulation of Notch1 and SOX2 and the upregulation of vimentin^[71]^.

The poor prognosis of GBM patients and the high levels of radio- and chemoresistance are strictly associated with the highly infiltrative capacities of these tumors. Primary GBM comprises both proneural and mesenchymal GSCs, but the treatment causes proneural to mesenchymal transition, determining the prevalence of mesenchymal GSCs in resistant tumors^[72]^. The mechanisms are analogous to those underlying the epithelial–mesenchymal transition (EMT). SLUG expression, which is linked to the mesenchymal profile, is regulated by the Notch1 pathway and has been implicated in radioresistance^[73]^. An elegant study demonstrated that GBM-derived neurosphere cultures with elevated Notch levels were prone to form intracranial tumors with major infiltrative characteristics. In this scenario, elevated Notch1 expression confers a more invasive phenotype to neurosphere cultures once matched to the low Notch1 experimental condition^[74]^. Downregulation of urokinase-type plasminogen activator/urokinase-type plasminogen activator receptor (uPA/uPAR), a multifunctional system playing a critical role in GBM invasion, inhibited the cleavage of the Notch receptor, thus inhibiting Notch signaling-induced AKT, NF-κB, and extracellular signal-regulated kinases (ERK) pathways^[75]^.

## TARGETING NOTCH SIGNALING IN THE EMERGENCE OF RESISTANCE TO CURRENT GLIOBLASTOMA THERAPIES

The existence of the GSC population within the tumor is an important limiting factor for achieving therapeutic success for GBM patients. These GSCs possess increased resistance to chemo- and radiotherapy compared to bulk cancer cells, although they appear to be sensitive to treatment targeting Notch^[57,76]^. However, additional difficulties limit the therapeutic response of GBM patients, such as insufficient drug delivery across the blood–brain barrier, abundant intra- and intertumoral heterogeneity, and an immunosuppressive microenvironment. The therapeutic resistance in GBM is particularly complicated by the interplay between GSCs and the TME, which is crucially orchestrated by the Notch pathway^[77]^, suggesting that the novel therapeutic approach targeting Notch could overcome drug resistance. In the subsequent sections, we review the state of our knowledge regarding the functional role of Notch signaling pathway in drug resistance and several approaches that are potentially valuable to overcome drug resistance for the efficacious treatment of gliomas.

### Direct and alternative targeting of Notch signaling

Starting from the molecular structure of Notch receptors, Notch ligands, and Notch activators, several studies have aimed to target the Notch pathway pharmacologically. The majority of identified compounds revealed anticancer activity in several preclinical studies^[78,79]^. Moreover, the combined effects of several inhibitors with components of the current therapeutic portfolio are still under investigation in different clinical trials. Since the stimulation of Notch depends mainly on γ-secretase complex activity^[80]^, γ-secretase inhibitors, inhibiting NICD from Notch receptors, are the most promising in different solid cancers including GBM^[81]^. The inhibition of the second cleavage of the Notch receptor is possible through α-secretase inhibitors, which block members of the disintegrin and metalloproteinase (ADAM) family^[82]^. Additionally, novel promising therapeutic tools that directly or indirectly target Notch signaling have been developed to overcome therapy resistance.

### Gamma secretase inhibitors

Several forms of γ-secretase inhibitors have been tested for anti-tumor effects, and DAPT is the first γ-secretase inhibitor tested in brain tumor experimental models^[83,84]^. DAPT stimulus in medulloblastoma xenografts induced apoptosis in cancer cells^[85]^. The positive effects of DAPT fortified the progress of γ-secretase inhibitors such as RO4929097, MRK-003, and L-685,458 (here indicated as GSIs)^[86]^.

GSIs have been utilized in clinical and experimental studies in many tumors. Most of these studies have assumed that GSIs, except for potency, are biologic equivalents. However, the biology of GSIs is more complex, and the general assumption of pharmacological and functional equivalency of GSIs needs to be investigated further. Different studies designate several GSIs as the most clinically relevant anti-Notch treatments^[87]^, even though the overall success in clinical trials shows limitations due to off-target complications^[88]^. These drugs have been analyzed on several subcutaneous brain tumor models, indicating the inhibition of neurosphere tumor growth by GSI-18. In established glial tumor models, Notch signaling downregulation using a single compound had partial effects, but it improved the efficacy of DNA-interfering agents. Accordingly, DAPT has been shown to improve the efficiency of TMZ^[89]^. Furthermore, DAPT has been shown to improve the efficiency of TMZ *in vivo*, as a single agent and in combination. Further studies in glial tumor models evaluated the GSI RO4929097 activity *in vitro*. Cotreatment with RO4929097 and chemotherapeutic agent improved TMZ properties and potentiated the cytotoxicity of etoposide and cisplatinum in glial tumor models. RO4929097 enhanced irradiation effects in GBM xenografts and decreased tumor growth in advanced-stage GBM xenografts. The authors concluded that the described preclinical data are necessary for the clinical development of Notch inhibitors in glial tumors^[90]^. Combined therapy with GSI RO4929097, radiation, and TMZ increased the survival of orthotopic GBM mouse models. The triple combination is more active than radio- and chemotherapy or GSI alone^[91]^. Similarly, DAPT intensifies the effects of radiation and decreases GSC growth and the number of endothelial cells disrupting the perivascular niche^[92]^. DAPT, tested in combination with an EGFR inhibitor, reduced endothelial cell sprouting and downregulated VEGF secretion. Therefore, the concomitant targeting of Notch and EGFR enhances the inhibitory effects on GBM angiogenesis and cell viability, suggesting a supplementary valuation of this targeting approach in clinical settings^[93]^. Similar to DAPT, other compounds, such as benzyloxicarbonyl-leu-leu-Nle-CHO (LLNle), induce GSC death and proteolytic stress and reduce NICD amount^[94]^.

Furthermore, *in vivo* studies have shown that in the absence of supplementary therapies, less invasive tumors are generated from neurospheres isolated by xenografts previously treated with MRK003^[95]^. Similarly, a tripeptide with γ-secretase inhibitor activity may target CD133+ cells at a low concentration to serve as a radiosensitizer for GBM patients.

Some clinical trials have been directed to estimate the tumor-suppressive effects of a limited number of Notch inhibitors in recurrent invasive gliomas^[88]^. However, the lack of activity by RO4929097 and a marked reduction of steady-state drug levels have been described^[96]^. The cause for the absence of activity needs to be clarified. A possible mechanism could be the auto-induction of RO4929097 metabolism. A phase 0/I study evidenced the effect of chemo-radiotherapy in combination with RO4929097 in newly diagnosed GBMs. It found the reduction of cells expressing CD133, a putative GSC marker. The authors concluded that, although evidence of target modulation was observed, recurrence occurred, associated with alterations in angiogenesis signaling pathways and variable blood–brain barrier penetration^[79]^. In a phase I/II trial, the combination of RO4929097 with bevacizumab targeting VEGF in patients with progressive or recurrent malignant glioma was tested. The drug combination was well-tolerated, and GBM patients indicated complete or partial remission after radiography^[97]^.

### α-secretase inhibitors

Notch is first cleaved by α-secretase outside the plasma membrane, via ADAM-10 and -17. A potent α-secretase inhibitor tested in GBM decreased cell proliferation and tumor size and prolonged survival *in vivo*. Furthermore, combination with DAPT reduced HES1 and HEY1 levels as well as leukemia inhibitor factor (LIF) and chitinase 3-like 1 (YsKL-40) levels, two new key players in GBM pathogenesis^[82]^.

### Antibodies

In addition to the secretase inhibitors, other molecules have been used to block Notch signaling in GBM, in order to overcome the drug resistance which drives disease progression. Blocking antibodies targeting specific isoforms of Notch receptors and engaged in contrast to the NRR, as well as blocking the receptor folding permitting ADAM cleavage, has been employed in preclinical and *in vitro* studies^[98]^. The specific antibodies, such as the anti-Notch1, proposed for GBM patients show the advantage of fewer side effects than GSIs. Another group of antibodies blocks Notch receptor - ligand interactions by hampering the EGF repeats essential for binding^[99]^. The first-in-human tested humanized anti-Notch1 blocking antibody treatment in a collection of GSC models with elevated Notch1 expression counteracted the transcription of Notch pathway target genes and caused significant impairment of cellular invasion under chemotherapy. However, the authors concluded that the observed phenotype could be due in part to Notch1 blockage as well as the activation of off-target signals^[100]^. Further pharmacological models were directed to Notch ligands such as for anti-Dll4 treatment. A rise in vessel density and reduced tumor dimension were observed after the administration of recombinant Dll4-Fc or anti-Dll4 polyclonal antibody in a bevacizumab-resistant human fibrosarcoma tumor model^[101]^. Similar findings were obtained in the human GBM cell line U87, subcutaneously implanted in nude mice. The authors showed that the overexpression of dominant negative soluble Dll4ECD-Fc enhanced the number of blood vessels and reduced tumor growth *in vivo*^[102]^. Anti-Dll4 antibodies are now being used in clinical trials.

### Natural agents

The empirical screening of thousands of molecules permits identifying compounds with potential antineoplastic effects within the available GBM preclinical experimental models. The Notch signaling pathway is the target of several natural compounds, and although the exerted molecular mechanisms are not completely understood, they have been tested for therapeutic aims. Resveratrol (RSV), a well-known polyphenol^[103,104]^, may reverse multidrug resistance, and several reports demonstrate that it can act as a sensitizer in different types of tumor cells to standard therapeutic agents^[105]^. Resveratrol inhibited HEY1 levels and NCID in GBM cells. Moreover, we found that low doses of RSV and GSI cotreatment resulted in the induction of GBM cell apoptosis and the concomitant block of the autophagic flux. We showed a prolonged rise of microtubule-associated protein light chain 3 (LC3-II) and p62 expression levels, related to a marked decrease of cyclin-dependent kinase 4 (CDK4), an important regulator of lysosomal function. The activation of autophagy after RSV and GSI stimulus, when the cells have impaired lysosomal function, caused the collapse of the system and thereafter apoptosis. The current studies addressed how the Notch inhibitor and RSV combination could be prospectively implemented in a novel therapeutic strategy for GBM treatment^[81]^. In A172 and T98G GBM cell lines with a heterozygous p53 mutation, RSV restored wild-type p53 expression, and these effects were mediated by the stimulation of Notch1 levels in a time-dependent manner. Inhibition of AKT and B-cell lymphoma 2 (BCL-2), increase of BCL2-associated X (BAX) expression, and cleavage of caspase 3 were observed, indicating the great pro-apoptotic effect of RSV^[106]^. Some *in vitro* and *in vivo* studies have shown that alpinetin, a natural chalcone distributed in a wide range of plants, can suppress the proliferation and invasiveness of GSCs by suppressing Notch signaling^[107]^. Diosgenin, a natural steroidal sapogenin, induces the differentiation of GBM cells, as shown by the increase of GFAP protein levels and decrease of N-MYC, telomerase reverse transcriptase (TERT), and Notch1^[108]^. Additionally, falcarindiol, a cytotoxic and anti-inflammatory polyacetylenic oxylipin, has anticancer properties against GBM cells by evoking the differentiation of GSCs, as well as triggering the apoptosis pathway. Conversely, the authors showed that falcarindiol has detrimental effects by disrupting the maintenance of normal neural stem cells and changing the balance between self-renewal and differentiation by suppressing forkhead transcription factor family O (FoxO), which preserves the adult stem cell population through the Notch axis^[109]^.

### Other agents

Antihelmintic niclosamide is a previously unrecognized candidate for clinical development. Niclosamide led to cytostatic, cytotoxic, and antimigratory effects, strongly reducing the frequencies of multipotent/self-renewing cells *in vitro* and *in vivo*. Mechanism of action analysis revealed that niclosamide simultaneously inhibited intracellular signaling, including Notch, mTOR, and NF-κB signaling cascades. Additionally, deletion of the nuclear factor kappa-b inhibitor alpha (NFKBIA) locus, encoding the inhibitor of the NF-κB and EGFR signaling pathway, could be a biomarker that predicts the synergistic activity of niclosamide with TMZ for GBM therapy^[110]^. Several studies consider the idea of peptides inhibiting intracellular Notch signaling. A peptide that mimics MAML1 binding to NICD–RBPJ has been shown to disrupt the ternary transcription complex and pass through the cell membrane^[111]^.

## CONCLUSIONS AND FUTURE DIRECTIONS

This review highlights the compelling body of evidence that demonstrates the pivotal role of Notch signaling in coordinating mechanisms of therapy resistance in high-grade brain tumors. The molecular basis of therapeutic resistance in GBM is complex and multifaceted due to the dynamic interplay between the tumor cells, GSCs, TME, and different aberrant signaling, including Notch signalling. Recent studies have evidenced that Notch signaling exerts a dynamic action sustaining the complex interaction among different cell types composing TME, thereby driving GBM recurrence and therapy resistance. Furthermore, redundant signaling pathways converging on Notch signaling can determine increased stem cell population, expression of multidrug transporters, and alteration of DNA repair activity, which control the emergence of disease progression. Besides, the worse prognosis of GBMs and the high levels of radio- and chemoresistance are strictly connected with the highly infiltrative capacities of these tumors, which are partially Notch pathway-dependent. Therefore, the successful treatment of GBM may rely on combinations of therapies, acting on multiple specific targets, converging on Notch inhibition, which could overcome drug resistance. The design of drug dosing schedules and delivery methods to improve brain penetration, together with a better understanding of GBM heterogeneity and Notch orchestrated signaling network, would assure the best possible performance when the treatments are used in combination therapy settings. However, further studies are necessary to ascertain the role of Notch as a predictive biomarker of acquired chemoresistance to be implemented in molecular screening strategies that would be crucial for identifying patients who might benefit from specific treatment combinations.

## DECLARATIONS

### Copyright

© The Author(s) 2022.
